# Application of MRI-Based Radiomics in Preoperative Prediction of *NF2* Alteration in Intracranial Meningiomas

**DOI:** 10.3389/fonc.2022.879528

**Published:** 2022-09-28

**Authors:** Shuchen Sun, Leihao Ren, Zong Miao, Lingyang Hua, Daijun Wang, Jiaojiao Deng, Jiawei Chen, Ning Liu, Ye Gong

**Affiliations:** ^1^ Department of Neurosurgery, Huashan Hospital, Fudan University, Shanghai, China; ^2^ Institute of Neurosurgery, Fudan University, Shanghai, China; ^3^ Shanghai Key Laboratory of Brain Function Restoration and Neural Regeneration, Shanghai, China; ^4^ Department of Neurosurgery, Changhai Hospital, Naval Medical University (Second Military Medical University), Shanghai, China; ^5^ Department of Critical Care Medicine, Huashan Hospital, Fudan University, Shanghai, China

**Keywords:** meningioma, radiomics, *NF2*, machiene learning, SVM - support vector machine

## Abstract

**Purpose:**

This study aimed to investigate the feasibility of predicting *NF2* mutation status based on the MR radiomic analysis in patients with intracranial meningioma.

**Methods:**

This retrospective study included 105 patients with meningiomas, including 60 *NF2*-mutant samples and 45 wild-type samples. Radiomic features were extracted from magnetic resonance imaging scans, including T1-weighted, T2-weighted, and contrast T1-weighted images. Student’s t-test and LASSO regression were performed to select the radiomic features. All patients were randomly divided into training and validation cohorts in a 7:3 ratio. Five linear models (RF, SVM, LR, KNN, and xgboost) were trained to predict the *NF2* mutational status. Receiver operating characteristic curve and precision-recall analyses were used to evaluate the model performance. Student’s t-tests were then used to compare the posterior probabilities of NF2 mut/loss prediction for patients with different NF2 statuses.

**Results:**

Nine features had nonzero coefficients in the LASSO regression model. No significant differences was observed in the clinical features. Nine features showed significant differences in patients with different NF2 statuses. Among all machine learning algorithms, SVM showed the best performance. The area under curve and accuracy of the predictive model were 0.85; the F1-score of the precision-recall curve was 0.80. The model risk was assessed by plotting calibration curves. The p-value for the H-L goodness of fit test was 0.411 (p> 0.05), which indicated that the difference between the obtained model and the perfect model was statistically insignificant. The AUC of our model in external validation was 0.83.

**Conclusion:**

A combination of radiomic analysis and machine learning showed potential clinical utility in the prediction of preoperative NF2 status. These findings could aid in developing customized neurosurgery plans and meningioma management strategies before postoperative pathology.

## Introduction

Meningioma is the most common primary tumor of the central nervous system (CNS), accounting for approximately 26.1-38.3% of all intracranial tumors (1–3). According to the WHO CNS tumor grading criterion, meningiomas are categorized into three grades and 15 histological subtypes based solely on the morphological features of the tumor cells. Despite the widespread use of the WHO classification, it fails to accurately predict the clinical behavior, aggressiveness, and recurrence of particular tumors. With the deeper understanding of the molecular landscape of meningioma, in addition to the histological diagnosis, the newest 2021 CNS tumor diagnostic criterion began to integrate the molecular and genetic profiling to assist in diagnoses and evaluate prognosis.

The *NF2* gene was first implicated in meningiomas after it was found that its inactivation resulted in the genetic tumor predisposition syndrome of neurofibromatosis type 2. *NF2* is a tumor suppressor gene comprised of 17 exons with 2 splicing isoforms that is positioned on chromosome 22q12.2 (4). Alterations in the NF2 gene, which can be caused by mutation, allelic inactivation, splicing alterations, or Chromosome 22 loss, have been implicated in approximately 30-60% of sporadic meningiomas, making it the single most frequent gene alteration in this tumor (5). The frequency of NF2 mutations is simlar in WHO Grade 1, 2 and 3 grades. However, it varies among histological subtypes and locations and are more likely to be observed in atypical and cerebral hemispheres. NF2 gene inactivation is considered to play a significant role in the development of meningiomas (5, 6). Patients with *NF2* mutations were also reported to show worse outcomes (7). Clinical trials targeting NF2 has been under way (NCT02523014). Thus, prediction of the *NF2* status before surgery can aid in the development of personalized treatment strategies for meningioma patients.

Radiomics is a novel practice in the field of machine learning. It could be used to extract and analyze medical imaging data (8). By conversion of sparse magnetic resonance imaging (MRI) into data, an immense amount of imaging information that is otherwise invisible to the naked eye in multiple dimensions could be generated (9). Radiomics is a potential approach for noninvasive high-throughput mining of tumor characteristics and has been applied in several other intracranial tumors, including glioma and schwannoma (10, 11). For meningiomas, algorithms have been developed in previous studies to predict WHO grade, tumor texture, peritumoral edema, and Ki-67 labels through radiomics. These models reported good performance in terms of accuracy and sensitivity (12–14). The status of well-known genetic changes could be accurately predicted by radiomics in several CNS tumors. However, such studies were scarcely mentioned in meningiomas (15, 16).

In this study, we investigated the utility of a radiomics signature based on multiparametric MRI as a preoperative and noninvasive biomarker of NF2 status in meningiomas.

## Materials and Methods

### Patients

A total of 105 meningioma patients underwent surgical resections between 2019 and 2021 at Huashan Neurosurgical Center were enrolled. Histological diagnoses were reviewed according to 2016 WHO meningioma grading criteria by two experienced neuropathologists (Dr. H.C and Dr. HX.C). Clinical information including age, gender, location, treatment status, the extent of resection, surgical outcome, and neurological functions was extracted from the medical records. Patients with recurrent meningioma who underwent another opration to remove the recurrent tumor were considered as recurrent meningioma cases. Patients with multiple meningiomas were also recorded. The clinical data of 105 patients was shown in.

**Table 1** 30 meningioma patients from First Affiliated Hospital of Nanjing Medical University were enrolled as External verification. The clinical data of 30 patients are shown in **Supplementary Material 1**. The specific research process of this study is shown in **Figure 1**. This study was approved by the Human Subjects Institutional Review Board of Huashan Hospital, Fudan University.

**Figure 1:**
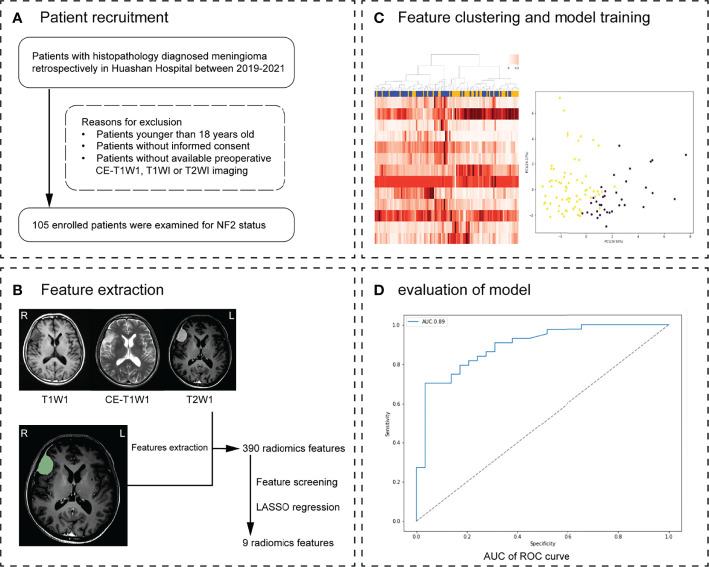
Workflow. **(A)** Patient recruitment strategy. **(B)** 390 features were extracted from region of interest (ROI) on each magnetic resonance imaging (MRI) sequence. **(C)** The inner loop included hyperparameter tuning and features selection in the training datasets. After feature selection, the model with optimal parameters was used for prediction in the test set. This procedure developed 10 different models with specific sets of features and hyperparameters. **(D)** The effectiveness of the model was verified in the validation group. Receiver operating characteristic (ROC) analysis and precision and recall (P-R) analysis were used for model performance evaluation. The MRI scans of 30 meningioma patients from another hospital were used as external validation.

**Table 1:** Clinical data of enrolled patients.

	NF2 mut/loss (60)	NF2 wild (45)	All (105)	P
Age	54.10 ± 9.90	51.93 ± 9.14	53.17± 9.60	0.254
Female/Male	2.33	2.21	2.28	1.00
WHO grade				
WHO grade 1 WHO grade 2 WHO grade 3	50 (64.29%) 9 (28.57%) 1 (4.08%)	40 (13.21%) 5 (79.25%) 0 (7.55%)	90 14 1	0.47
Location				
Skull base Convexity Parasinoidal	20 (33.33%) 14 (23.33%) 26 (43.33%)	25 (55.56%) 7 (15.56%) 13 (28.89%)	45 (42.86%) 21 (20.00%) 39 (37.14%)	0.07
Multiple	3	0	3	0.258
Recurrent	12	4	16	0.170
Ki-67 labeling index(%)	4.10 ± 2.70 (range1-12)	3.67 ± 1.94 (range1-8)	3.91 ± 2.40 (range1-12)	0.341
PR positive H3K27me3 positive	46 (76.67%) 51 (85.00%)	40 (88.89%) 39 (86.67%)	86 (81.9%) 91 (86.7%)	0.130 0.773

### Next Generation Sequencing

Bidirectional sequencing was performed to detect microlesions in the *NF2* gene. DNA was extracted from tumor tissue with TIANamp Genomic DNA Kit (Tiangen Biotech, Beijing, China) as instructed by the manufacturers. The whole coding sequence and the exon-intron boundaries of the gene were amplified by a standard polymerase chain reaction (PCR). Subsequently, the product was used for bidirectional sequencing, as described previously (17). The sequence data were analyzed by Sequencer 4.9 (Genecode, MI, USA) and compared with the *NF2* sequence (NM_016418) from GenBank. Mutations were described according to the standard nomenclature for DNA sequence changes according to the Human Genome Variation Society (HGVS).

### Loss of Heterozygosity Analysis of 22q

Each tumor sample was subjected to PCR analysis. A fraction of the PCR product (0.5 liters) was mixed with 0.1 liters of Genescan 500 size standard (PE Applied Biosystems Foster City, CA, USA) and 0.9 liters of formamide loading buffer. Combinations were eletrophoresed on a 5 percent polyacrylamide gels on an ABI 377 DNA sequencer (PE Applied Biosystems Foster city CA, USA) for 2 hours after being denaturated at 96°C for 5 minutes. Individual gel lanes were visualized using the Genotype 2.0 software. The samples were scored using strict criteria. The two highest peaks within the predicted size range were designated as alleles. A loss of heterozygosity was defined as a ratio of T1:T2/N1:N2 of less than 0.67 or more than 1.50. The majority of normal DNA amplifications yielded two PCR results, showing heterozygozity. The ratio of allelic loss to informative instances was used to calculate the LOH frequency of a locus. The average LOH frequency of the long arm of chromosome 22 was the sum of the LOH frequencies of each location.

### Immunohistochemistry

Immunohistochemical staining was performed using monoclonal antibodies against Ki-67 [Signalway (SAB), Shanghai, China; 1:200 dilution]. The cells stained in immunohistochemistry accounting for more than 10% of all cells were considered positive(+), otherwise negative (–). Progesterone receptor (PR) level was also examined to classify the tumors into two categories: PR negative (–) or PR positive(+). H3K27me3 was examined with anti-H3K27me3 (Millipore, 07-449) on ECL Plus films (Carestream).

### MRI Image Acquisition

All patients underwent MRI scanning before operation (with or without Gadolinium enhancement). MRI scans were performed by the Trio 3.0-T scanner (Siemens, Erlangen, Germany). The imaging process included axial T1WI (TE, 15 ms; TR, 450 ms; slice thickness, 5 mm), T2WI (TE, 110 ms; TR 5800 ms; slice thickness, 5 mm), and CE scans using 0.1 mM/kg gadopentetate dimeglumine (TE, 15 ms; TR, 450 ms; slice thickness, 5 mm). Tumor location was described according to Al-Mefty’s published manuscript, such as parasagittal/falx, skull base, cerebral convexity, etc.

### Tumor Segmentation and Feature Extraction

Preprocessing was performed using the 3D-Slicer software (version 4.11). The MRI DICOM files of all patients were imported into 3D-slicer. T1WI, T2Flair, and DWI images were registered to the T1C sequence images; N4 bias field correction was applied to each sequence image to correct non-uniformities in intensity. Two neuroradiologists painted regions of interest (ROIs) on T1c images using the 3D-slicer software. Multiple meningiomas from the same patient were considered as a single case in ROI classification and impact feature extraction. Enhancement of the dural tail sign was included in ROI, while peritumoral edema was excluded. The neuroradiologists were not informed of the clinical and biomarker data. Pyradiomics, an open-source python package (https://github.com/Radiomics/pyradiomics), was used to extract radiomic features.

### Feature Selection and Establishment of Prediction Model

Pyradiomics, an open-source python package (https://github.com/Radiomics/pyradiomics), was used to extract radiomic features from the ROIs of each patient’s images. After that, we eliminated the time, the checked hospital, machine model and other useless information. 130 radiomics features were retained in each sequence. To avoid degradation in model performance due to overfitting and increase in feature dimension, we evaluated radiomics features in distinguishing between mut/wild type and screened all radiomic features of each sequence to generate a new feature set.

First, three pairs of samples in two groups were tested using t-test analysis. Levene test was used to test the homogeneity of variance. For the data with the homogeneity of variance greater than 0.05, a t-test was used to detect whether the characteristics of the average of the two groups of independent samples showed significant differences (P < 0.05). Only characteristics with significant differences were retained.

Second, a further feature screen was performed based on LASSO regression, which added L1 regular expression based on the least square regression. The features screened by t-test were standardized; the optimal parameter lambda was selected after ten-fold cross-validation. Thus, the corresponding coefficients of the model were trained. Features with nonzero coefficients in the LASSO regression model were selected.

### Model Training and Evaluation

Machine learning models were developed to predict the outcome of NF2 status based on different algorithm. We used five supervised machine learning algorithms to establish the prediction model. All the cases were randomly divided into training and validation cohorts in the ratio of 7:3. The algorithm was trained based on the training group, and its effectiveness was verified in the validation group. The algorithm with the highest AUC (area under the curve) in the validation cohorts was chosen as the best model. Five prediction models were generated by random forest (RF), k-nearest neighbor (KNN), support vector machine (SVM), logistic regression (LR), and extreme gradient boosting (xgboost) methods. The training and validation cohorts were divided based on the selected feature subset. Predictions were made after iterative optimization. The sensitivity, specificity, accuracy, and F1 score were evaluated. The model performance was analyzed by plotting ROC (receiver operating characteristic), P-R (analysis and precision-recall), and calibration curves. The MRI scans of 30 meningioma patients from First Affiliated Hospital of Nanjing Medical University were used as external validation to verify the accuracy of the best model.

### Statistical Analysis

Statistical analysis was performed using SPSS 26.0 (IBM SPSS statistics 26.0 for mac; IBM corp). For continuous variables, the Student t-test was used; for comparison of mean values of continuous variables, ANOVA was used. Categorical variables were compared using the χ2 test and the Fisher test. Continuous data were expressed as mean ± SD. P-value <0.05 was considered statistically significant.

## Results

### Clinical Data and Immunohistochemistry

A total of 105 patients (32 males and 73 females) with intracranial meningiomas were recruited in the training and testing cohorts, including 60 patients with NF2 mutation/loss and 45 wild type patients. The mean age of the patients was 53.17 ± 9.60 years (range 31 to 73 years). There are 90 (85.71%) grade 1, 14 grade 2 (13.33%) and 1 grade 3 patients (0.95%), respectively. The most common pathological subtype was fibrous (45, 42.86%). The median Ki-67 labeling index was 3.91 ± 2.40(range 1-12). PR was positive in 86 patients (81.9%). Loss of H3K27me3 expression was observed in 14 patients (13.3%). No diffence was observed of the compared the clinical and immunohistochemical characteristics between *NF2* mutant and wild-type groups. Parasinoidal (26/60) was the most common location in *NF2* mut/loss group, while *NF2* wild tumours were more likely in skull base locations (25/45). Three cases of multiple meningiomas were identified in our cohort. However, only one tumor in each patient was removed, which were all belonged to the *NF2* mutation group. 16 patients (4 with wildtype *NF2* and 12 with *NF2* mutations) were regarded as recurrent meningiomas and no difference was observed between *de novo* and recurrent patients regarding NF2 status (p = 0.170).

### *NF2* Sequencing Analysis and LOH Analysis

Among all 105 patients, 52 patients (49.52%) had *NF2* mutations; 8 patients (7.62%) showed loss of *NF2* gene due to partial deletion of chromosome 22q. The remaining 45 patients had wild-type *NF2*. Allelic deletion of *NF2* and mutations were classified as NF2 mutation group, amounting to 60 cases. Of all patients with *NF2* mutations, 23 were nonsense mutations; 16 were frameshift mutation; 8 were splice site mutation; 4 were missense mutation. Exon 1 and Exon 6 was detected the highest mutation frequency, accounting for 6 (11.54%) and 7 (13.46%) of all mutants, respectively. However, we did not find any obvious hot spot mutations. The most common copy number deletion occurred in 22q11.21- q13.33. Details of the *NF2* mutation status were shown in **Supplementary Table 1**.

### Radiomic Feature Selection and Radiomic Signature Construction

**Figure 2:**
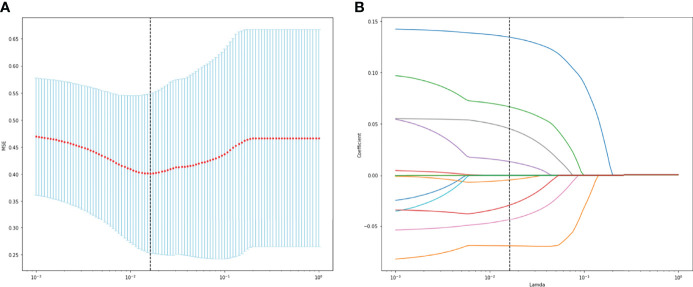
**(A)** The change of MSE corresponding to the LASSO method. **(B)** Lamda value screening of LASSO regression.

**Figure 3:**
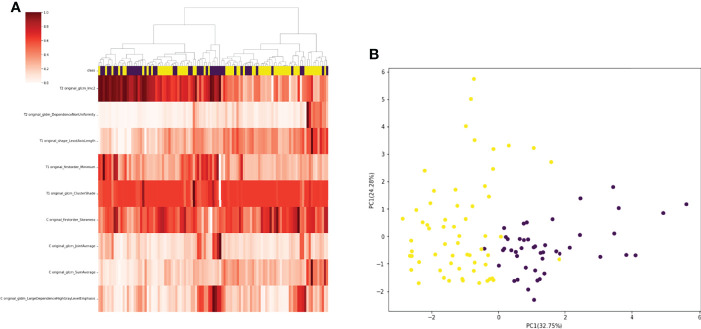
**(A)** 105 patients with meningiomas were divided into two categories by hierarchical cluster analysis. **(B)** PCA (Principal Component Analysis) plot showing the distribution of principal components of the radiomics features. The majority of NF2-mut meningioma and NF2-wild meningioma cases were spatially separated.

A total of 130 radiomic features were extracted from each sequence. 390 radiomic features were included in the screening process. 147 radiomic features showed statistically significant differences between the *NF2* mut/loss and wild-type groups. Only 9 features had nonzero coefficients in the LASSO regression model. The screening process of Lamda is shown in **Figure 2**. In **Figure 2A**, The red line represents the standard deviation of the mean square error (MSE) of λ. The blue bar indicates the range of the mean square error. The lambda with the lowest standard deviation is the most suitable for classification and the model is the simplest. Therefore, We chose the position with the lowest lambda standard deviation (red line) as the most appropriate λ Value. The details and p-values for these 9 features are shown in **Table 2**. 4 features were from the CE-T1Flair, 3 from T1WI, and 2 from T2WI sequences. 7 features could describe the texture of tumors and 2 described the wavelet of tumors. The 9 radiomics features were selected for the model building. We tried to cluster nine radiomics features through unsupervised hierarchical cluster analysis. PCA (Principal Component Analysis) is used to reduce the dimension and describe the distribution of data (**Figure 3**).

**Table 2:** The details of selected radiomics features.

Name	Sequence	Type	p
glcm_Imc2	T2	Texture	0.027
gldm_DependenceNonUniformity	T2	Texture	0.022
shape_LeastAxisLength	T1	Wavelet	0.037
firstorder_Minimum	T1	Texture	0.025
glcm_ClusterShade	T1	Texture	0.037
firstorder_Skewness	CET1	Wavelet	0.001
glcm_JointAverage	CET1	Texture	0.005
glcm_SumAverage	CET1	Texture	0.005
gldm_LargeDependenceHighGrayLevelEmphasis	CET1	Texture	0.005

Finally, we decided to incorporate the 9 features into the model. Gray Level Dependence Matrix (GLDM) quantifies gray level dependencies in an image. included descriptors of the three-dimensional size and shape of the ROI. First-order statistics describe the distribution of voxel intensities within the image region defined by the mask through commonly used and basic metrics.

Gray Level Co-occurrence Matrix (GLCM) describes the second-order joint probability function of an image region constrained by the mask.

### Model Training and Performance

**Figure 4:**
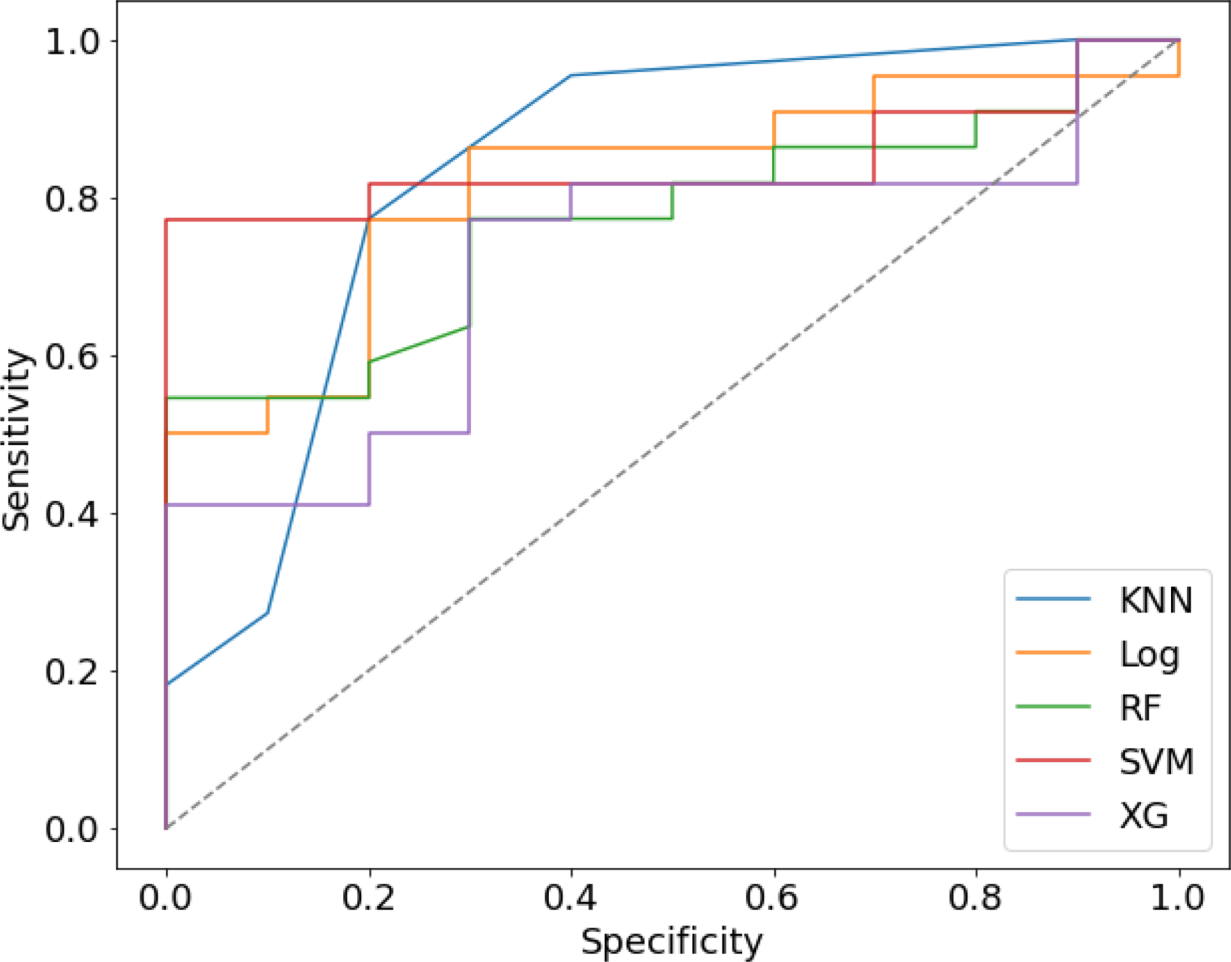
The Receiver operating characteristic (ROC) curve of five prediction models in validation cohort.

We randomly divided all patients into training and validation groups in a ratio of 7:3. 73 patients were included in the training group. 9 radiomic features of 73 patients were used to train 5 supervised machine learning algorithms (RF, SVM, LR, KNN, and xgboost). The data of a total of 32 patients in the validation cohort was used to evaluate the effectiveness of the algorithms. The AUCs of each algorithm in training and validation cohorts were calculated and compared as shown in **Table 3** and **Figure 4**. SVM (support vector machine) had the highest AUC of 0.89 in the training cohort and 0.85 in the validation cohort. F1 score is the harmonic average of accuracy and recall, while the value was 0.80 in the SVM model. LR (logistic regression) had an AUC of 0.84 in the training cohort and 0.82 in the validation cohort. Most of the algorithms had AUCs above 0.7.

**Table 3:** The performances of five prediction models.

Comparisons	Cohorts	LR	KNN	Xgboost	SVM	RF
AUC	train	0.85	1	1	0.89	1
	test	0.85	0.76	0.82	0.85	0.77
Sensitivity	train	0.775	1	1	0.893	0.806
	test	0.75	0.692	0.74	0.7	0.6
Specificity	train	0.781	1	1	0.737	0.765
	test	0.8	0.789	0.88	0.727	0.765
Accuracy	train	0.779	1	1	0.779	0.779
	test	0.781	0.751	0.78	0.71	0.688
F1-score	train	0.729	1	1	0.685	0.716
	test	0.72	0.692	0.76	0.609	0.643

### Internal and External Verification

**Figure 5:**
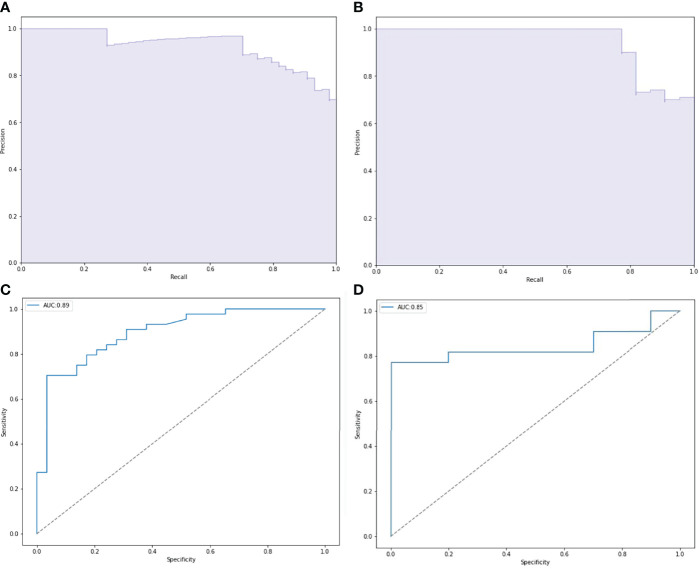
Performance of *NF2* status predictive models based on SVM. **(A, C)** Receiver operating characteristic (ROC) curve and precision-recall (P-R) curve of SVM predictive model in training group. **(B, D)** ROC curve and P-R curve of SVM predictive model in validation group.

**Figure 6:**
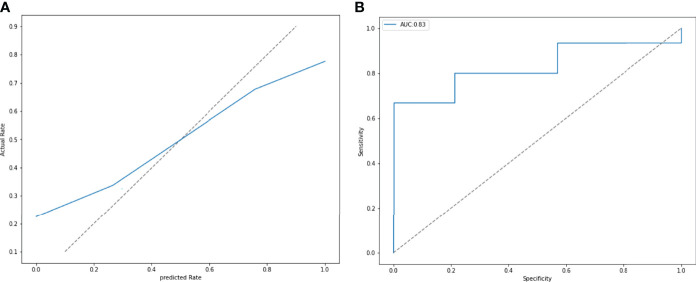
**(A)** The calibration curve analysis and Hosmer-Lemeshow test for SVM model demonstrated the observations and predictions in validation cohorts were in good accordance. (P = 0.411). **(B)** External validation was performed by 30 patients from other hospitals. The SVM model had an AUC of 0.83.

**Figure 7:**
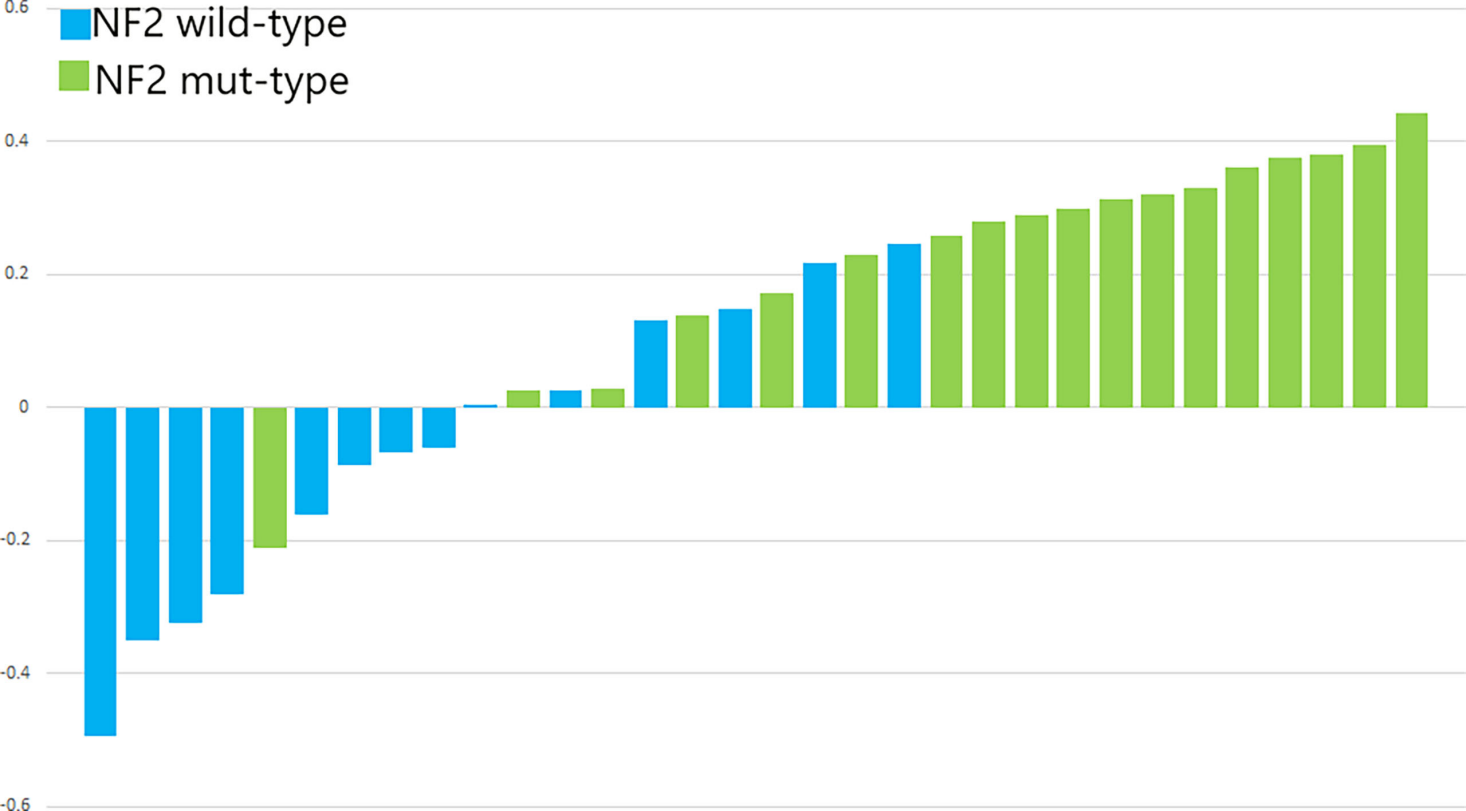
The p-values of SVM for the validation cohorts. The blue bars show the radiomics signature values for the *NF2*-wild meningiomas, and the green bars show the values for the *NF2*-mut meningiomas.

ROC and P-R curve analysis of SVM in training and validation cohorts were shown in **Figure 5**. AUC of SVM were 0.89 and 0.85 in the training cohort and validation cohort respectively. **Figure 6** shows the predicted value and their actual mutation of each validation group sample. Samples with a predicted value greater than 0 were predicted to be NF2 mut-type by the SVM model. The actual mutation of the sample were showen by color, Green represents mut-type and blue represents wild-type. 30 meningioma patients from First Affiliated Hospital of Nanjing Medical University were enrolled as external validation to verify the accuracy of the SVM model. the AUC of SVM model is 0.82 in the external validation cohorts. The calibration curve analysis and Hosmer-Lemeshow test for SVM model demonstrated the observations and predictions in validation cohorts were in good accordance (**Figure 7**).

## Discussion

*NF2* inactivation was the most common alteration in meningiomas and played an important role in tumor progression (5, 18). Given this, the prediction of *NF2* inactivation status before surgery might be meaningful for determining an appropriate personalized treatment strategy. In this study, we built a machine learning model to preoperatively predict the status of *NF2* inactivation by radiomic analysis. We observed that the models based on SVM produced excellent results in the machine-learning experiments. Our radiomics model may aid the early identifcation of meningioma patients with *NF2* mutation.

*NF2* is located on the long arm of chromosome 22 (chr22q) and encodes a 69 kDa protein named merlin (moesin-ezrin-radixin-like protein). NF2 is a member of the Band 4.1 FERM gene family (19). Merlin plays important role in several essential pathways, including HIPPO pathway, mTOR/PI3K/AKT pathway, and receptor tyrosine kinases (RTKs) (20, 21). Previous studies showed that more than 60% of sporadic meningioma patients harbored somatic mutation, epigenetic inactivation, or allele loss of *NF2* on chr22q. The proportion is even higher in high-grade meningiomas (22–24). In a study of 88 sporadic meningiomas, 49% exhibited allelic loss of chromosome 22, 24% had *NF2* somatic mutations and 26% had aberrant *NF2* promoter methylation. In 17% of the meningiomas, epigenetic *NF2* inactivation was the only cause of *NF2* deficiency (24). Compared to *NF2*-wt meningioma, *NF2* mutant meningiomas was detected with a higher proliferation index (Ki-67 labels) and often manifested in comparatively larger tumor size (25). In addition, the deletion of *NF2* leads to overexpression of focal adhesion kinase (FAK), resulting in enhanced cell migration and invasion (26). In all, non-invasive preoperative prediction of *NF2* mutation might be of use. The knowledge of NF2 status might play a role in decision making of appropriate clinical treatment strategies for meningioma patients.

Recently, several studies on radiomics focused on meningiomas and demonstrated encouraging results. Previous studies could distinguish WHO grade I meningiomas out of WHO grade II and III meningiomas by radiomic analysis models. These models were proved to have high accuracy and sensitivity (27, 28). Lei et al. distinguished two subtypes in WHO grade I meningiomas by radiomics with an accuracy higher than 90% (29). Other studies focused on predicting clinical characteristics of meningiomas, such as extent of peritumoral edema and tumor consistency. For example, Bing et al. analyzed peritumoral edema in meningioma patients using an SVM-based machine learning algorithm combined with clinical data (14). Zhai et al. constructed a radiomic-based signature to predict meningioma consistency with AUC of 0.94 in the validation cohort (13). Taken together, previous studies proved the feasibility of radiomic analysis for meningioma imaging. Some studies also predicted the molecular typing of other primary tumors, such as breast cancer and glioma. Monti et al. extracted quantitative radiomic features from DCE-MRI pharmacokinetic data to differentiate ER, PR, and HER2 status in breast cancer (30). In glioma, radiomics had been used to predict IDH mutation and co-deletion of 1p/19q (31, 32). However, up to now, the report of such studies on meningiomas is scarce.

In this study, we first detected the occurrence of *NF2* inactivation in the tumor samples. Mutations or loss of *NF2* gene were also considered as inactivation of *NF2*. By feature extraction and screening, we finally obtained 9 significant radiomic features. In previous studies, screening features of meningiomas ranged between 3 to 22 (27–29). The discrepancy might be attributed to difference in the process of screening and imaging data heterogeneity. Most of the 9 features were extracted from T1WI plain scan and Gadolinium enhanced sequences (33). The AUC of linear model based on SVM was 0.85 and 0.82 in the internal and external validation cohorts respectively, consistent with findings of other studies which predicted intracranial tumor biomarkers (31, 32). There were many radiomics studies involving HER2 in breast cancers and IDH1 in gliomas (34, 35). Approximately, 70% of HER2 mutations occured between amino acids 755 and 781 (exons 19 and 20) in breast cancers (36). 80% of IDH1 mutations in gliomas occured on R132H (37). *NF2* has 17 exons and harbors no significant hotspot mutations (21). Our cohorts also showed no significant hotspot mutations, in accordance to previous reports. This may affect our prediction results; however, we found AUCs for most of our models were above 0.7 in the validation cohort. The data was not sufficient enough to distinguish the differences between *NF2* mutation types.

In our cohort, multiple meningiomas in a single patient were considered as one tumor in ROI selection and feature extraction. That’s because they chose to remove only the symptomatic meningioma. Somatic mutation of *NF2* is related to neurofibromatosis type 2 (38) and is often found in multiple intracranial meningiomas. All our 3 cases of multiple meningiomas belonged to the *NF2* mutation group. Whereas, the difference in number of multiple meningiomas between *NF2* mutant and wild-type groups had no statistical significance. *NF2* plays an important role in progression of meningiomas (39). Additionally, there was no significant difference in number of relapse patients between the two groups, The limitations mentioned above might be attributed to the comparatively small sample size. A study by Clark et al. reported that in meningiomas with *NF2*- mutations, tumor location had a predilection for the posterior and lateral skull base, tentorium, and cerebral falx, while sporadic mutations, such as those in TRAF7 and SMO, tended to be relevant with anterior skull base location (5). This phenomenon was not observed in our cohort. In some previous studies, clinical data were added to radiomic models to optimize the impact (14). No significant clinical features were found in our cohort, so these were not included in the analysis.

Recently, radiomics studies of other diseases selected ROI through automatic segmentation, such as lung cancer, breast cancer and gastric disease (40–42). Jonathan et al. had developed an algorithm based on convolutional neural network to automatically segment vestibular schwannoma and achieved satisfactory results (43). The application of automatic segmentation could benefit our research and clinical practice. Although at present, no research showed that there is a difference in accuracy between automatic segmentation and manual segmentation. Some studies showed that there was no meningioma tumor cell in the gadolinium enhanced meningeal tail sign (44), while some others drew the opposite conclusion (45). We included the meningeal tail sign in ROI analysis because the boundary between the meningeal tail sign and meningioma is difficult to distinguish.

There were limitations in our present study. First, this was a retrospective study with comparatively small sample size which could have limited the accuracy of our model. Probably due to restriction of sample size, many difference in clinical and immunohistochemical features showed no statistical significance. Secend, the result of this study predicts binary variables. All our radiomic algorithms were based on linear models. There might be nonlinear models with a higher fitting degree. Finally, the clinical follow-up of these patients is still underway. Hopefully, the follow-up data might further confirm the significance of preoperative prediction of *NF2* status.

## Conclusion

This retrospective study demonstrated that multiparametric MRI-based radiomics analysis could be a promising approach for preoperative prediction of *NF2* inactivation in patients with meningioma. It could serve as an effective non-invasive approach to predict *NF2* inactivation and help determine individualized therapeutic regime for patients with meningioma.

## Data Availability Statement

The original contributions presented in the study are publicly available. This data can be found here: https://www.ncbi.nlm.nih.gov/sra, PRJNA858222.

## Ethics Statement

Written informed consent was obtained from the individual(s) for the publication of any potentially identifiable images or data included in this article.

## Author Contributions

SS prepared the first version of the editorial. LR, ZM, LH and YG discuss the editorial content and revised the final editorial text. All authors contributed to the article and approved the submitted version.

## Acknowledgments

This study was supported by grants from the National Natural Science Foundation of China (82072788 to YG, 82203390 to LYH and 82203204 to JJD), the Science and Technology Commission of Shanghai Municipality (22140900200 to YG) and Shanghai Sailing Program (20YF1403900 to LYH).

## Conflict of Interest

The authors declare that the research was conducted in the absence of any commercial or financial relationships that could be construed as a potential conflict of interest.

## Publisher’s Note

All claims expressed in this article are solely those of the authors and do not necessarily represent those of their affiliated organizations, or those of the publisher, the editors and the reviewers. Any product that may be evaluated in this article, or claim that may be made by its manufacturer, is not guaranteed or endorsed by the publisher.
