# Supplementary material for: Application of MRI-Based Radiomics in Preoperative Prediction of NF2 Alteration in Intracranial Meningiomas
Source: Front Oncol. 2022 Sep 28;12:879528. doi: 10.3389/fonc.2022.879528 (PMC9578175; doi:10.3389/fonc.2022.879528)
Supplement: Supplementary file 1 [file Table_1.docx]

|  | NF2 mut/loss (16) | NF2 wild (14) | All (30) |
| --- | --- | --- | --- |
| Age | 55.81±7.99 | 51.71±12.46 | 53.90±10.34 |
| Female/Male | 4.33 | 3.67 | 4 |
| WHO grade  WHO grade 1  WHO grade 2  WHO grade 3 | 15(93.75%)  0  1(6.25%) | 13(92.86%)  1(7.14%)  0 | 28(93.33%)  1(3.33%)  1(3.33%) |
| Location  Skull base  Convexity  Parasinoidal | 5(31.25%)  7(43.75%)  4(25%) | 6(42.86%)  7(50%)  1(7.14%) | 11(36.67%)  14(46.67%)  5(16.67%) |
| Ki-67 labeling index(%) | 3.50±2.25（range1-8） | 3.86±1.99(range1-8) | 3.91±2.40(range1-12) |
| PR positive  H3K27me3 positive | 11 (76.67%)  12 (85.00%) | 10 (88.89%)  11 (86.67%) | 21(81.9%)  23(86.7%) |

**Supplementary material 1** Clinical data of 30 externally validated patients
