# Supplementary material for: Application of MRI-Based Radiomics in Preoperative Prediction of NF2 Alteration in Intracranial Meningiomas
Source: Front Oncol. 2022 Sep 28;12:879528. doi: 10.3389/fonc.2022.879528 (PMC9578175; doi:10.3389/fonc.2022.879528)
Supplement: Supplementary file 2 [file Table_2.docx]

|  | NF2 mut/loss (16) | NF2 wild (14) | All (30) |
| --- | --- | --- | --- |
| Age | 54.10 ±9.90 | 51.93 ±9.14 | 53.17±9.60 |
| Female/Male | 2.33 | 2.21 | 5 |
| WHO grade  WHO grade 1  WHO grade 2  WHO grade 3 | 50 (64.29%)  9 (28.57%)  1 (4.08%) | 40 (13.21%)  5 (79.25%)  0 (7.55%) | 90  14  1 |
| Location  Skull base  Convexity  Parasinoidal | 20(33.33%)  14(23.33%)  26(43.33%) | 25(55.56%)  7(15.56%)  13(28.89%) | 45(42.86%)  21(20.00%)  39(37.14%) |
| Multiple | 3 | 0 | 3 |
| Recurrent | 12 | 4 | 16 |
| Ki-67 labeling index(%) | 4.10 ±2.70（range1-12） | 3.67±1.94(range1-8) | 3.91±2.40(range1-12) |
| PR positive  H3K27me3 positive | 46 (76.67%)  51 (85.00%) | 40 (88.89%)  39 (86.67%) | 86(81.9%)  91(86.7%) |

**Supplementary materials 2** Clinical data of 30 patients with external validation
